# Biosorbent Based on Poly(vinyl alcohol)–Tricarboxi-Cellulose Designed to Retain Organic Dyes from Aqueous Media

**DOI:** 10.3390/polym15030715

**Published:** 2023-01-31

**Authors:** Ramona-Elena Tataru-Farmus, Ramona Cimpoesu, Iulia Nica, Daniela Suteu

**Affiliations:** 1 Department of Chemical Engineering, “Cristofor Simionescu” Faculty of Chemical Engineering and Environmental Protection, “Gheorghe Asachi” Technical University of Iasi, Prof. Dr. docent D. Mangeron Blvd., No. 73A, 700050 Iasi, Romania; 2 Department of Materials Science, Faculty of Materials Science and Engineering, ”Gheorghe Asachi” Technical University of Iasi, Prof. Dr. docent D. Mangeron Blvd., No. 41, 700259 Iasi, Romania; 3 Department of Organic, Biochemical and Food Engineering, “Cristofor Simionescu” Faculty of Chemical Engineering and Environmental Protection, “Gheorghe Asachi” Technical University of Iasi, Prof. Dr. docent D. Mangeron Blvd., No. 73A, 700050 Iasi, Romania

**Keywords:** aqueous medium, biosorption, cationic organic dye, cellulose, hydrogel as biosorbent

## Abstract

Methylene Blue, a cationic dye, was retained from aqueous solutions using a novel biosorbent made of poly(vinyl alcohol) reticulated with tricarboxi-cellulose produced via TEMPO oxidation (OxC25). The study of the Methylene Blue biosorption process was performed with an emphasis on operational parameters that may have an impact on it (such as biosorbent concentration, pH of the aqueous media, and temperature). The current study focused on three areas: (i) the physic-chemical characterization of the biosorbent (scanning electron microscopy (SEM) and energy-dispersive X-ray spectroscopy (EDX)); (ii) biosorption data modeling to determine the quantitative characteristic parameters employing three equilibrium isotherms (Langmuir, Freundlich, and Dubinin–Radushkevich—DR); and (iii) the study of temperature influence. The results of the study showed that the Langmuir model provided a good fit for the experimental data of biosorption, realizing a maximum capacity of 806.45 mg/g at 20 °C. The free energy of biosorption (E) evaluated by the DR equation was in the range of 6.48–10.86 KJ/mol. The values of the thermodynamic parameters indicated an endothermic process because the free Gibbs energy ranged from −9.286 KJ/mol to −2.208 KJ/mol and the enthalpy was approximately −71.686 KJ/mol. The results obtained encourage and motivate the further study of this biosorption process by focusing on its kinetic aspects, establishing the biosorption’s controlled steps, identifying the mechanism responsible for the retention of textile dyes presented in moderate concentration in aqueous media, and studying the biosorption process in a dynamic regime with a view to applying it to real systems.

## 1. Introduction

Industrialization and urban development inevitably attracted the development of industrial sectors and modern technologies, which caused an intensification of environmental problems, especially those involving water pollution [1,2,3]. Water is an essential natural resource, vital for maintaining the quality of life and ecosystems [1,4]. Although it is a gift of nature, it is a limited resource; the existing fresh water sources do not meet the needs of the current population [1,5].

Several industries are involved in water pollution, such as the chemical, pharmaceutical, textile, car production, mining industry, along with agriculture, household, and administrative activities—which pollute water with significant amounts of dyes, heavy metals, nitrates, phosphates, fertilizers, pesticides, surfactants, phytotoxins, organic solvents, and other chemicals involved in everyday life [1,5,6,7].

Due to the vital function that water plays in the development of living organisms (metabolism, agriculture, and industry), its pollution represents one of the most important environmental problems, as it represents a threat to ecosystems and the quality of life on Earth [8].

A variety of industries employ industrial dyes, such as textile, leather, paper, cosmetics, the plastics industry, and the food industry. According to the research, they are among the most significant/problematic water pollutants [4,5,9,10,11]. The majority of synthetic dyes are toxic, carcinogenic, poorly biodegradable, stable to light and heat, highly soluble in water, rapidly transferred to water, accumulate in living cells, and more [1,12,13,14,15].

On the market, there are more than 100,000 different types of dyes, and more than 7.105 tons of dyes are mass-produced each year worldwide [15,16]. According to expert studies, approximately 2% of these synthetic dyes are lost during industrial processing steps, and 15–25% of the yearly worldwide dye production is misplaced in the coloring process and dumped into the water without additional treatment [4,10,11,12,14]. The release of dye effluents from dye production or consumption units into water bodies has an environmental effect on the receiving water systems. This can cause serious health problems for humans, plants, and animals, as well as aquatic biota. It also damages the aesthetic appearance of the waters [1,4,5,10].

The current challenges that researchers are facing are related to the design of dye-removal methods under the constraints of the imposition of very tight legal requirements for the removal of dyeing effluents in wastewater [9].

Since dye retention is a concern and threat, the development of efficient and economical removal techniques from wastewaters is ongoing (an important category of polluting organic compounds) and is crucial for the ecosystem [4,12].

There are four types of wastewater treatment technology: physical, chemical, biological, and electrochemical [5]. Ion exchange, precipitation, reverse osmosis, adsorption, flocculation/coagulation, oxidative processes, and membrane filtration are physical and chemical methods used to treat industrial wastewater containing dyes [15,17,18,19,20,21]. These approaches, however, have a variety of drawbacks in terms of cost, viability, influence on the environment, effectiveness, operational difficulty, handling of toxic by-products, etc.

Adsorption is still one of the methods that is frequently used to retain some chemical pollutants, mainly because it offers benefits that are hard to ignore, such as the ability to use a variety of inexpensive adsorbent materials (also known as “low cost” adsorbents) regardless of the type of pollutant and the necessary process conditions. Different biomaterials can be employed as biosorbents in adsorption techniques used to remove contaminants from water and wastewater because of their adsorbent qualities (e.g., dyes, phenol, pesticides, toxic metal ions, drug residues) [22,23,24,25,26,27,28,29,30,31,32,33,34]. In order to effectively and affordably remove dyes from wastewater, biosorption can be used as a sustainable wastewater treatment method [35,36,37].

One of the major benefits of the adsorption process is represented by the possibility of using as adsorbents an extremely wide range of materials with sorption properties, from synthetic materials (ion exchange resins, functionalized cellulose, activated carbon, synthetic polymers), to cheap, natural materials, as well as waste from various industries and agriculture (natural lignocellulosic materials (husks, tree bark, leaves), algae, or residual microbial biomass, etc.) [32,33,38,39,40,41,42].

The use of green materials has increased in recent decades. Both the process design and the use of a larger variety of materials obtained from the processing of renewable resources have seen an acceleration in this field. Their novelty and diversity are a consequence of their nature, structure, and specific properties, but also of the type of processing process according to the intended applications. These applications are expanding more and more and are based both on the use of techniques for valorizing some resources represented by industrial by-products or on innovative approaches (derivatizations, the creation of multicomponent assemblies and supramolecular architectures, hybridizations, additions, post-processing in various commercial forms, etc.) on already known material [20].

Due to their adsorbent properties, biomaterials based on renewable resources can be used as biosorbents in biosorption processes applied in environmental protection, aimed at removing chemical pollutants (dyes, phenol, pesticides, toxic metal ions) from water, soil, and air based on the formation of physical and/or chemical bonds between the pollutant and the adsorbent [1,30,32,33,43,44,45]. Among the polysaccharides of interest in obtaining biosorbents used in environmental protection, we can mention cellulose, starch, dextran, chitin and chitosan, agarose, and alginic acids. Polysaccharides, in their natural form or after physical and/or chemical treatments, show adsorptive properties, utilizable in concentration–separation–purification processes as biosorbents with high specificity [46,47,48,49,50,51,52].

A relatively new and particularly interesting and challenging category of biomaterials with adsorbent properties resulting from the processing of renewable resources is represented by *hydrogels*. A new class of adsorptive biomaterials consists of polymeric structures having hydrophilic and hydrophobic fractions in a specified proportion, convenient water-swelling capacity, hydrophilicity, biocompatibility, and low toxicity. These characteristics encourage their use in a number of fields, such as those involved in environmental protection, ensuring people’s health, in agricultural, or industrial activities [53,54,55,56].

To obtain hydrogels, different polysaccharides or renewable resources can be used as the basic units subjected to the crosslinking process, the best-known being cellulose, chitosan, poly(vinyl alcohol), and pullulan [26,57,58,59].

Hydrogels have thus attracted growing interest for the treatment of wastewater due to their high adsorption capacities, regeneration capabilities, and potential for reuse in ongoing technological processes, water-swelling capacity, high specific surface area, and a variety of surface-active functional groups [52,53,56,60,61]. These biomaterials also have a variety of drawbacks, namely ones relating to low strength, stability, and mechanics. Numerous technological processes, including crosslinking techniques using auxiliary agents (i.e., crosslinking agents), have been researched and developed in order to increase the biosorptive capabilities [52,53,54,60].

Poly(vinyl alcohol) (PVA), a biodegradable polymer utilized recently to create hybrid biodegradable biomaterials, has been the subject of a number of investigations. Although it has poor mechanical strength, several elements that could increase it have been explored. Research has focused on specific natural polymers (cellulose and pullulan) in various concentrations, in the form of oxidized C6 derivatives, as important multifunctional components that could serve as crosslinking agents as well as agents for enhancing the hardening and stiffness properties of hybrid PVA hydrogels [52,53,60,61,62,63]. These biosorbents derived from natural resources can also be employed to successfully retain some heavy metal species from various aqueous systems.

This study’s objective is to study the biosorptive properties of an innovative biosorbent made of hydrogel (OxC25) which is based on a matrix of poly(vinyl alcohol) (PVA) cross-linked with tri-carboxy cellulose. The goals of this biosorption research were to (i) identify operational parameters that affect the biosorption process and their appropriate values; (ii) model the experimental data utilizing three of the most widely used adsorption isotherms and thermodynamic analysis; and (iii) physically–chemically characterize the biosorbent before and after it retained the cationic dye, Methylene Blue.

## 2. Materials and Methods

### 2.1. Materials

*Biosorbent.* A hybrid hydrogel was prepared from 2,3,6-tricarboxy-cellulose and poly(vinyl alcohol) (PVA), which was obtained by oxidizing wet cellulose pulp in the presence of a 2,2,6,6-tetramethyl-1-piperidinyloxy (TEMPO)/NaBr/NaOCl system. This biomaterial was created by “P. Poni” Institute of Macromolecular Chemistry, Iasi, Romania. PVA with MW = 8.9 104–9.8 104 g/mol, 99% hydrolyzed, and cellulose (microcrystalline, Avicel^®^ PH 101) with a degree of polymerization (DP) of 135 are the two basic components and were purchased from Sigma-Aldrich Co [61]. Pure chemical reagents of a commercial grade, TEMPO, sodium bromide, and 9% (wt) sodium hypochlorite, were used (Sigma Aldrich Co., St. Louis, MO, USA).

**Figure 1:**
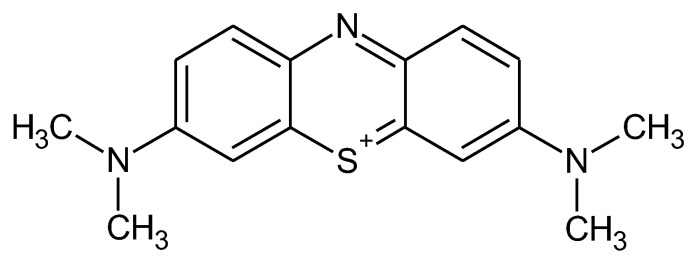
Chemical structure of cationic dye, Methylene Blue—C.I. 52015.

*Adsorbate*. A cationic dye, Methylene Blue (MB) (MW = 320 g/mol, λ_max_ = 660 nm, was chosen as a chemical pollutant (reference model of cationic dye) for this study with the chemical structure depicted in Figure 1. Methylene Blue (MB) is an organic dye belonging to heterocyclic aromatic compounds and is used in the medical area due to its antiseptic, analgesic, and disinfectant effect (as 1% solution) and in the industrial area as a synthetic dye for textiles [43].

The stock solution was made using a commercial dye with an initial concentration of 320 mg/L. The working solutions were made using double-distilled water and diluted according to the prescribed concentrations.

### 2.2. Methods

#### 2.2.1. Preparation and Physical–Chemical Characterization of Hybrid Hydrogel

**Figure 2:**
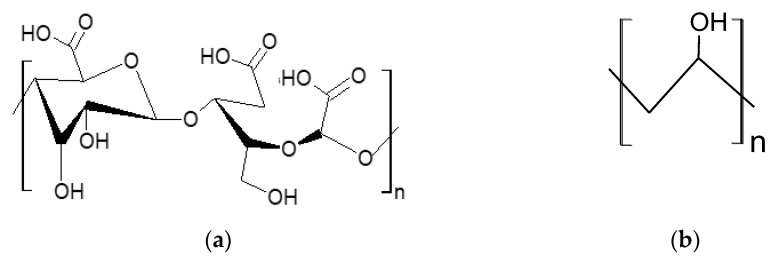
Chemical structure of the compound used to obtain OxC25 hydrogel: (**a**) 2,3,6 tricarboxy-cellulose; (**b**) poly(vinyl alcohol).

The specialized literature indicates various protocols for obtaining hydrogels based on 2,3,6 tricarboxy-cellulose (Figure 2a) and poly(vinyl alcohol) (PVA) (Figure 2b) [62,64]. The OxC25 hydrogel utilized as biosorbent in this investigation was made using the procedure and guidelines provided in our prior work [63].

#### 2.2.2. Batch Biosorption Methodology

Studies on experimental biosorption were carried out in batch settings, using various quantities of biosorbent and 25 mL of cationic dye solution with starting concentrations between 12.8 and 83.2 mg/L. The selected temperature (5 °C, 20 °C, and 50 °C) was maintained using a cooler (for 5 °C) and a thermostatic oven Poleko SLW53 model (Pol-Eko-Aparatura sp.j., Wodzisław Śląski, Poland) (for 20 °C and 50 °C). Using 1N HCl and/or 1N NaOH solution, the pH levels were adjusted to the predetermined values.

Considering the maximum dye wavelength of 660 nm and a Shimadzu UV-1280 UV–VIS spectrophotometer (Shimadzu Corporation, Kyoto, Japan), the dye concentration of the equilibrium solution was measured spectrophotometrically in accordance with Lambert–Beer law. The biosorption experiments were conducted in triplicate. Equation (1) was used to calculate the biosorption capacity specific to the studied biosorbent (q, mg of dye/g of biosorbent). Equation (2) was used to calculate other biosorption parameter, R% (the percent of dyes removal):

(1) $$q=\frac{C_{0}-C}{G}\cdot V$$

R% = [(C_0_ − C)/C_0_] × 100 (2)

where C_0_ and C are the dye initial and the equilibrium concentration in solution (mg/L), G is the amount of biosorbent (g), and V is the volume of solution (L).

#### 2.2.3. Physical–Chemical Characterization of Biosorbent

*Lyophilization*. Freeze-dried samples were used for biosorbent characterization investigations. The lyophilization operation, which was carried out with a Labconco lyophilizer (Labconco, Kansas City, MO, USA), was performed as per following operational parameters: 0.05 mBar pressure, temperature of −50 °C, for a 6 h period.

*Scanning electron microscopy (SEM) and Energy-dispersive X-ray (EDX).* Microstructural and chemical analyzes, before and after the biosorption procedure, were performed using a VEGA-TESCAN LMH II, Scanning Electron Microscope (Tescan Orsay Holding, Brno—Kohoutovice, Czech Republic) with SE detector, WD 15.5 mm, 30 kV, HV, and equipped with EDS detector, Bruker Nano GmbH, Berlin, Germany. For mapping distribution of elements, Esprit 2.2 software (Bruker AXS Microanalysis GmbH, Berlin, Germany) was used in automatic mode. For quantitative chemical analysis, the calculation of weight and atomic percentages % was performed using an average of 10 values and Standard Deviation was calculated for each element.

#### 2.2.4. Modeling the Biosorption Experimental Data

Among the many models that describe the adsorption processes presented in the specialized literature, we selected three, considered more representative for the purpose pursued in our study [62,63,65]. Their general equations and the linearized forms are presented below:

- Freundlich (F)

(3) $$\text{general equation:}\;q=K_{F}\cdot C_{}^{1/n}$$

(4) $$\text{linearized equation:}\;\log q=\log K_{F}+\frac{1}{n}\log C_{}$$

- Langmuir (L)

(5) $$\text{general equation:}\;q=\frac{K_{L}\cdot C\cdot q_{0}}{1+K_{L}^{}\cdot C}$$

(6) $$\text{linearized equation I:}\;\frac{1}{q}=\frac{1}{q_{0}}+\frac{1}{K_{L}\cdot q_{0}}\cdot \frac{1}{C}$$

(7) $$\text{linearized equation II:}\;\frac{C}{q}=\frac{1}{q_{0}\cdot K_{L}}+\frac{C}{q_{0}}$$

- Dubinin–Radushkevich (DR)

$$q=q_{0}\exp\left(-B\cdot \varepsilon^{2}\right)$$

ln q = ln q_0_ − Bε^2^ (8)

$$\varepsilon =\mathrm{RT}\;\ln\left(1+\frac{1}{C}\right);\;E=\frac{1}{\sqrt{2B}}$$

where: K_F_ and 1/n—are the Freundlich parameters related to the biosorption capacity and intensity (efficiency), respectively; a favorable biosorption corresponds to a value of 1 < n < 10; q_0_ and K_L_ are Langmuir constants, q_0_ is the maximum amount of retained dye (mg/g) and K_L_ is the constant related to the binding energy of dye (L/mg); q_D_ is the maximum biosorption capacity (mg/g); β is the activity coefficient related to mean biosorption energy; ε is the Polanyi potential and E—the mean free energy of biosorption (kJ/mol).

### 2.3. The Study of the Thermodynamics of the Process

The Langmuir constant, K_L_, was determined at three different temperatures and the following equations were used to calculate three relevant thermodynamic parameters (3–5) [62,64,65].

(9) $$\ln\;K_{L}=\;-\;\frac{{\Delta H}^{0}}{\mathrm{RT}}+\frac{{\Delta S}^{0}}{R}$$

(10) $$\Delta G=-RT\ln K_{L}$$

where ΔG is Gibbs free energy variation (kJ/mol), ΔH is enthalpy variation (kJ/mol), and ΔS is entropy changes (kJ/mol K), R—the universal gas constant (8.314 J/mol K), T—the temperature of solution (K), and K_L_ is the value of Langmuir constant (L/mol).

### 2.4. Error Analysis

The linear regression coefficient, R2, is the most frequently used error function, despite the fact that many other error functions, including mean relative error, a hybrid error function, Marquardt’s percentage standard deviation, non-linear chi-square test, sum of squared errors, standard deviation of relative errors, sum of absolute errors, and Spearman’s correlation coefficient [66,67], have been used to determine the accuracy of many studies. The residual root-mean-square error (MSE) (6) and the chi-square test (7) were selected for this study:

(11) $$RMSE=\sqrt{\frac{1}{N-2}\sum_{i=1}^{N}\frac{{\left(q_{e,\exp}-q_{e,calc}\right)}^{2}}{q_{e,calc}}}$$

(12) $$\chi^{2}=\sum_{i=1}^{N}\frac{{\left(q_{e,\exp}-q_{e,calc}\right)}^{2}}{q_{e,calc}}$$

where q_e,exp_ and q_e,calc_ represent the experimental and calculated values of sorption capacity (mg/g), and N is the number of experimental data.

## 3. Results and Discussion

### 3.1. Biosorbent Analysis Using SEM and EDX

The typical surface morphologies and compositions were analyzed trough scanning electron microscopy for the characterization of different properties of the studied biomaterial. Furthermore, micrographs and elemental distributions are presented for each biosorbent sample.

**Figure 3:**
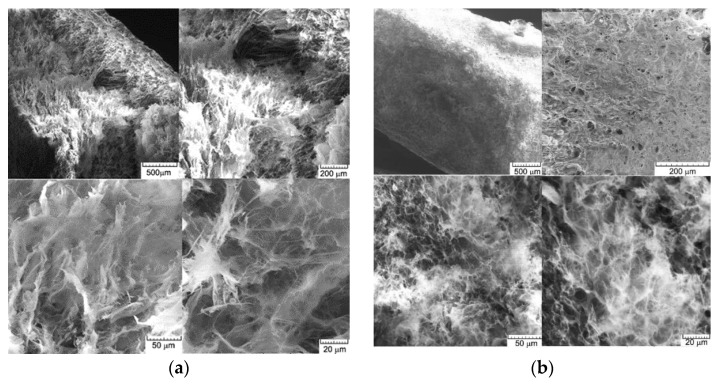
Scanning electron microscopy (SEM) of the biosorbent after (**a**) and before (**b**) Methylene Blue cationic dye biosorption.

The biosorbent was examined using *scanning electron microscopy* both before and after the biosorption of MB dye. Images obtained at 50, 100, 500, and 1000× magnifications are shown in Figure 3.

**Figure 4:**
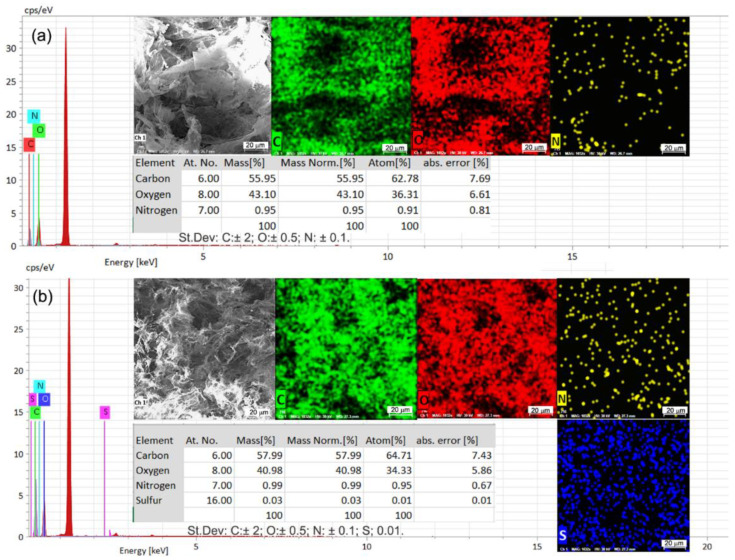
Energy-dispersive X-ray (EDX) spectra of the biosorbent OxC25 after (**a**) and before (**b**) Methylene Blue cationic dye biosorption.

Additionally, energy-dispersive X-ray spectroscopy was used to evaluate the chemical composition of the biosorbent material both before and after MB biosorption (EDX). The determinations were performed in three areas on the surface of the material (1 mm^2^) and an average was used. While the spectra analysis of the biosorbent prior to the biosorption of MB Dye (Figure 4a) identifies C, O, and N at different X-ray energies, the spectra of the biosorbent after the biosorption process took place (Figure 4b) reveals the presence of S in addition to C, O, and N. The same changes in the biosorbent material structure before and after MB dye absorption are revealed by the mapping elements comparison.

The weight and atomic percentages % analysis of the biosorbent material prior to and following the biosorption of MB dye reveals the change in the chemical structure as well, as the presence of a small percentage of S can be noted (0.03% weight percentage in the post-biosorption material). These additional changes recorded in the chemical composition of the biosorbent are valid indicators of the biosorption of the Methylene Blue dye.

### 3.2. The Value of Point of Zero Charge (pH_PZC_) for Hydrogel

The behavior of the biosorbent in interactions with the chemical species that need to be retained can be predicted using the electrical surface charge of an adsorbent material. This can be assessed according to the value of the zero load point (pH_PZC_).

Based on the method of Nouri and Haghseresht [68], in our previous study we determined the value of pH_PZC_ parameters is 5.4 [64]. For pH levels below pH_PZC_, it is anticipated that the biosorbent’s surface will be positively charged (as a result of protonation of the characteristic groups, such as carboxylic ones) at this value, permitting the biosorbent to react with anionic species through electrostatic interactions and hydrogen bonding. Additionally, due to the dissociation of the -COOH functional groups at pH values above pH_PZC_, the sorbent surface is negatively charged and able to interact with cationic species electrostatically or by anion-exchanges.

### 3.3. Modeling the Biosorption Equilibrium Process

The biosorption on an adsorptive material often involves a variety of interactions, which are highly impacted by the functional groups contained in their structure, biosorbent features, the chemical form of contaminants, and the experimental settings (such as initial pollutant concentration, solution pH, temperature, phases contact time).

#### 3.3.1. Assessment of a Some Operational Parameters Affecting the Biosorption Process

Understanding the interactions between operational parameters, biosorbent structural characteristics, and chemical species absorbed during the process is the first step in analyzing the biosorption process.

##### Influence of Solution pH

**Figure 5:**
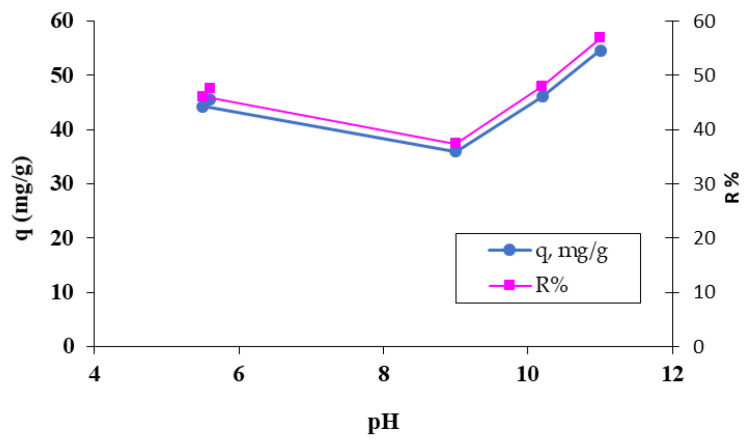
The influence of pH on the retention of the MB cationic dye on the OxC25 hydrogel. Operational conditions: C_0_ = 38.4 mg/L; 0.4 g/L biosorbent; T = 20 °C.

This parameter affects the performance of both phases involved in the biosorption process: the chemical species that must be retained from the aqueous medium (the MB dye) and the solid phase represented by the OxC25 hydrogel (the change in the degree of dissociation of the functional groups from the biosorbent structure that determines a certain surface charge). In this context, it is efficient to retain anionic or cationic species from the aqueous solution according to the pH value and the surface charge of the biosorbent (Figure 5).

According to Figure 5, the MB dye is biosorbed more efficiently from alkaline media, around pH 11, which can be seen both from the values of the amount (q, mg/g) and the percentage (R%) of MB dye adsorbed. This observation is in agreement with the fact that at a pH > pHPZC, the retention with maximum efficiency of the cationic species will be performed in a basic environment. The retention is achieved through electrostatic interactions, π–π interactions, and hydrogen bonds established between the dye molecules and the ionized functional groups, existing on the surface of the biosorbent.

##### Influence of the Biosorbent Concentration

**Figure 6:**
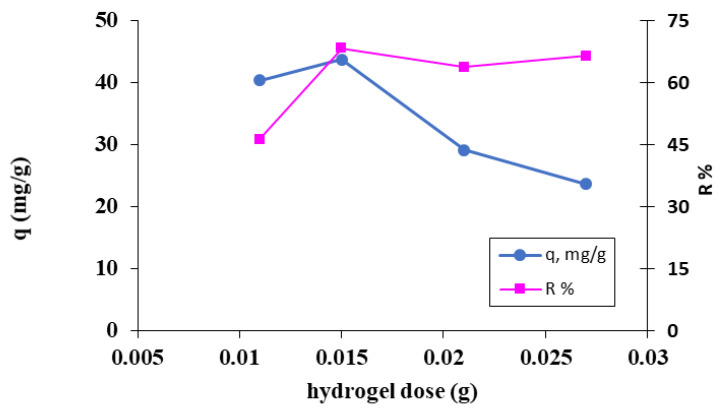
The influence of biosorbent dose on the retention of the MB cationic dye on the OxC25 hydrogel. Operational conditions: C_0_ = 38.4 mg/L; pH = 11; T = 20 °C.

Various quantities of tested hydrogel were combined with 25 mL of MB dye solutions of specific concentrations in order to determine the best value of biosorbent concentration. Figure 6 shows the results that were attained.

Figure 6 illustrates that with a biosorbent dose of 0.015 g, or 0.6 g/L, the highest retention is attained.

##### Influence of the Initial Dye Concentration and Temperature

**Figure 7:**
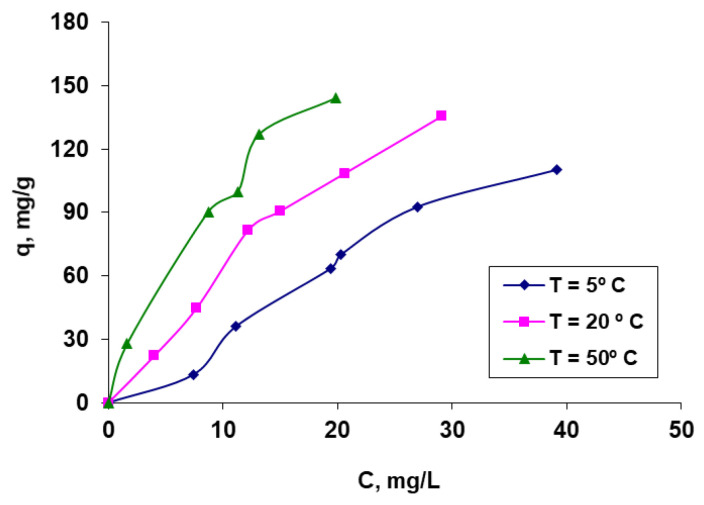
The biosorption isotherms of Methylene Blue dye on hydrogel OxC25. Conditions: pH = 11, contact time = 24 h, amount of biosorbent = 0.44 g/L.

From Figure 7, it is possible to observe first of all the behavior of the studied system at the variation of the initial dye concentration, as well as the influence of the variation of the temperature at which the biosorption process takes place. The biosorbent material reaches a state of saturation for a certain initial concentration of the dye that depends on the temperature at which the process takes place, after which the amount of retained dye tends to remain constant. The fact that the biosorption capacity values rise with temperature is another indication that the process may be endothermic in nature.

#### 3.3.2. Experimental Data Processing Based on Adsorption Isotherm Models

The amounts of the studied chemical species retained per gram of biosorbent at equilibrium (q, mg/g) as a function of its concentration at equilibrium (C, mg/L) in solution are represented graphically by biosorption isotherms.

Three isotherm models, presented in the scientific papers, were used to process the biosorption experimental data (Table 1), enabling quantification of distinctive parameters from the graphed form of their linearized equations (Table 1). The shape of the curves implies an “L” type of isotherm, subgroup 2, according to Gills’ classifications of isotherms [69]. These are, in fact, traditional Langmuir-type isotherms, which depend on the surface biosorption of molecules with a vertical orientation through exceptionally potent intermolecular interactions.

By looking at the organized data in Table 1, they may also underline some conclusions regarding the investigated biosorption process: for all three temperatures taken into account, the Langmuir 1 model best matches the experimental results (based on values for the correlation coefficient R^2^). One can make a preliminary observation about the mechanism of the biosorption process, namely whether it is physical or chemical, by taking into account the biosorption energy value (E) according to the DR model. Due to the electrostatic attraction created between the negatively charged surface of the biosorbent and the functional groups of the dye, the values obtained for E in the range of 6.482–10.859 kJ/mol are typical of biosorption processes based on physical bonds, such as van der Waals interactions, hydrogen bonds, and dipole–dipole interactions [70,71].

**Figure 8:**
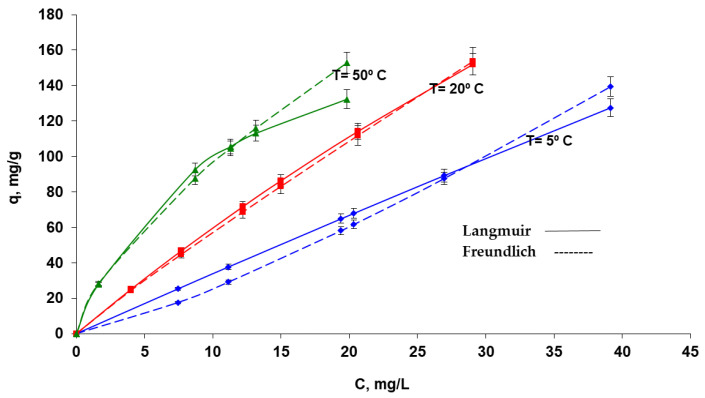
The biosorption isotherms (Langmuir I and Freundlich) of Methylene Blue dye on the hydrogel OxC25 biosorbent. Conditions: pH = 11, contact time = 24 h, amount of biosorbent = 0.4 g/L.

Figure 8 shows the isotherms obtained for the biosorption of the Methylene Blue dye onto OxC25 biosorbent, at the three considered temperatures, using the calculated values for the Langmuir and Freundlich isotherm parameters from Table 1.

The biosorption capacity value obtained at 20 °C was compared to other biosorption capacity values reported in the literature for other adsorption studies employing organic dyes from the aqueous medium on various types of hydrogels (Table 2).

The data presented in Table 2 show that the value of the biosorption capacity in the case of the studied hydrogel is comparable to other materials of the same time; that is, it can be considered a potential biosorbent for the retention of dyes from aqueous environments.

### 3.4. Thermodynamic Study

**Figure 9:**
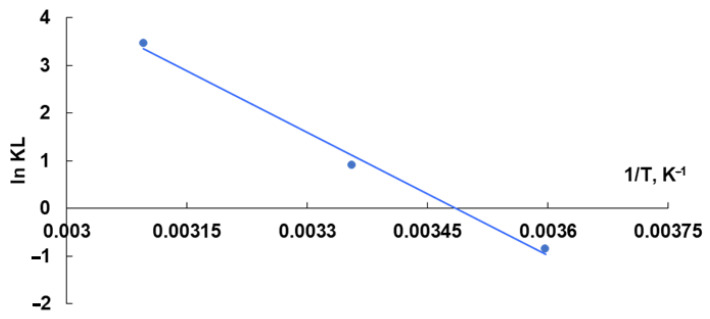
Graphical representation used to determine the ΔH and ΔS characteristic values of the biosorption process of Methylene Blue dye on OxC25 hydrogel as a biosorbent. Conditions: pH = 11, contact time = 24 h, amount of biosorbent = 0.4 g/L.

The following thermodynamic variables were thought to be more significant for the investigated biosorption process: ΔG^0^, ΔH^0^, and ΔS^0^. They were calculated based on Equation (3), as well as the relationships between the three parameters (4). Additionally, using the graphic representation lnK_L_ = f(1/T) with R^2^ = 0.993 (Figure 9), the value of ΔH^0^ and ΔS^0^ were determined [63,78].

Most of the negative values obtained for ΔG^0^ (Table 3) suggest that the biosorption of the cationic dye Methylene Blue on the tested biosorbent can be considered a spontaneous process at temperatures of 20 °C and 50 °C. The values obtained for ΔG^0^ between −20 and 0 kJ/mol suggest that a physical mechanism underlies the biosorption process. This information overlaps with the finding provided by the DR model that values of the mean free energy of adsorption (E) are in the range of physical biosorption.

The ΔH^0^ value (Table 3) is negative, indicating an endothermic process that is favorably impacted by temperature rise and the usage of energy to improve the degree of biosorption. The positive value of ΔS^0^ (Table 3) indicates that the dye molecules have a strong affinity for the biosorbent during the biosorption process and that there is a random increase at the biosorbent–adsorbate interface [78]. Additionally, the positive value of ΔS^0^ demonstrates that the dissociative mechanism serves as the foundation for the entire process [78].

## 4. Conclusions

The results of this work show that hydrogel OxC25-based biosorbents are effective at retaining organic dyes that are already present in aqueous solutions at moderate concentrations. The biosorption of the cationic Methylene Blue cationic dye was investigated using biosorbents based on the OxC25 hydrogel. The Langmuir, Freundlich, and Dubinin–Radushkevich isotherm models were used to process experimental data in order to evaluate the properties of the biosorbent, with the Langmuir model in this case offering the best fit to the data.

This study also demonstrated that the biosorption process using a biosorbent based on the OxC25 hydrogel for the removal of the Methylene Blue cationic dye is more physically than chemically driven. This is supported by the determined value of the free biosorption energy (E = 8.28–11.18 kJ/mol, from the DR model equation).

The process can be regarded as spontaneous and endothermic based on the negative values of the free energy Gibbs change (ΔG^0^ = −9.28 and −2.208 kJ/mol) and the positive value of the biosorption enthalpy change (ΔH^0^ = −71.686 kJ/mol).

All these results prove the biosorbent capacity of the hybrid hydrogel based on 2,3,6 tricarboxy-cellulose and poly(vinyl alcohol) (PVA) (namely OxC25) in the retention of organic dyes. 

The obtained results stimulate and motivate the deepening of the studies regarding this biosorption process, by approaching the kinetic aspects of the process, with the establishment of determining the biosorption’s controlled steps, the establishment of the mechanism involved in the retention of textile dyes, as well as the study of the biosorption process in a dynamic regime with a view to extending it to real systems.

Furthermore, taking into account all these findings, it can be said that biosorption using the OxC25 hydrogel as a biosorbent can be a feasible substitute solution for the retention of textile dyes in general, and cationic ones in particular, present in an aqueous environment under certain circumstances.

## Figures and Tables

**Table 1:** The Methylene Blue dye’s biosorption characteristics on a hydrogel-based biosorbent.

Isotherm	Temperature		
	5 °C	20 °C	50 °C
**Freundlich**			
K_F_ ((mg/g) (L/mg)^1/n^)	1.439 ± 0.743	6.727 ± 1.388	20.337 ± 1.727
n	0.802 ± 0.11	1.0768 ± 0.0792	1.481 ± 0.081
R^2^	0.932	0.972	0.991
χ^2^	10.779	5.592	1.879
RMSE	16.062	12.005	7.607
**Langmuir:**			
Langmuir I: (1/q = f (1/C))			
q_0_ (mg/g)	2557.54 ± 0.00094	806.45 ± 0.00139	199.203 ± 0.0003
K_L_ (L/g)	0.0013 ± 0.00145	0.00799 ± 0.0036	0.0997 ± 0.00386
R^2^	0.9903	0.9638	0.9987
χ^2^	2.089	3.7688	1.8322
RMSE	8.905	11.9792	8.6947
Langmuir II: (C/q = f (C))			
q_0_ (mg/g)	588.23	625.00	222.22
K_L_ (L/g)	0.0063	0.0104	0.0789
R^2^	0.448	0.443	0.943
**Dubinin–Radushkevich (DR):**			
q_0_ (mg/g)	23,711.99 ± 0.752	7532.23 ± 0.3397	2638.05 ± 0.1724
β (mol^2^ /kJ^2^)	0.0119 ± 0.00146	0.0076 ± 0.000547	0.0042 ± 0.0021
E (kJ /mol)	6.482 ± 0.4002	8.1 ± 0.016	10.859 ± 0.269
R^2^	0.943	0.9798	0.9927
χ^2^	−0.01902	−0.00547	−0.0016
RMSE	0.2057	0.1065	0.0559

**Table 2:** Achieved biosorption capacities while employing hydrogel as a biosorbent to retain dyes.

Biosorbent	Dye	Biosorption Capacity (mg/g)	Ref.
CaCO3@starch/polyacrylamide/TEMPO-oxidized nanocellulose, CaCO3@STA/PAM/TOCN	Congo red (CR) Methylene Blue (MB)	277.76/101.01	[72]
N-maleyl chitosan cross-linker	Methylene Blue and Crystal Violet	66.89/64.56	[73]
cross-linked chitosan hydrogels	Methylene Blue	1968	[74]
cross-linked chitosan hydrogels	Methylene Blue	1952	[75]
lanthanum-sodium alginate hydrogel	anionic azo-dyes: Direct Green 1 (DG 1) and Acid Blue 113 (AB 113)	909/983	[76]
calcium alginate hydrogel beads	Methyl Violet	1151	[77]
hybrid hydrogel from 2,3,6 tricarboxycellulose and poly(vinyl alcohol) (PVA) (OxC25)	Methylene Blue	806.45	this study

**Table 3:** Values of thermodynamic parameters characteristic of the biosorption process of Methylene Blue dye on OxC25 hydrogel as biosorbent.

T (K)	K_L_, L/g	ΔG^0^ (J/mol)	ΔH^0^ (kJ/mol)	ΔS^0^ (J/mol K)
278	0.00133	1974.305	-	-
293	0.00774	−2208.48	−71.686	249.852
323	0.09921	−9285.65	-	-

## Data Availability

The data presented in this study are available on request from the corresponding author.
